# Breaking the cycle of neglect: building on momentum from COVID-19 to drive access to diagnostic testing

**DOI:** 10.1016/j.eclinm.2023.101867

**Published:** 2023-02-16

**Authors:** Emma Hannay, Madhukar Pai

**Affiliations:** a FIND, Geneva, Switzerland; b School of Population and Global Health, McGill University, Montreal, Canada

Diagnostic testing is at the heart of quality healthcare. However, due to neglect of diagnostics, nearly half the world's population lacks access to essential tests.^1^ The Covid-19 pandemic has shone a harsh spotlight on inequities in access to testing. Only about 35% of tests administered worldwide have been used in low- and lower-middle- income countries (LMICs), where 75% of the global population lives.^2^ At the same time, the pandemic set off a boom in diagnostics.^3^ We have identified ten opportunities (Table) created by the pandemic that could be leveraged to drive better access to testing in general.

**Table:** Ten opportunities that can allow the global health community to improve access to diagnostics, building on the momentum of the Covid-19 pandemic.

1. Since the Covid-19 pandemic has increased public literacy around diagnostics, this could be used to demand better access to diagnostics for other conditions. 2. Political interest in diagnostics, surveillance and pandemic preparedness has increased. These create an opportunity to draw access to routine diagnostics up the priority list for future health reform and investment in health systems. 3. Increased use of digital companion tools to support community education and public health action has broader applications beyond Covid-19. 4. Self-testing and home-based testing became normalized for Covid-19, and could inspire self-testing or home-based sample collection for other conditions. 5. All countries have expanded molecular diagnostics capacity. This investment is a valuable platform that can be redeployed to address other diseases. 6. Rapid leap forward in the pipeline for innovative, point of care multiplex molecular platforms could play a vital joint role in both improving clinical care and building effective surveillance systems through their data sharing capabilities. 7. Rapid increase in gene sequencing capacity in low-and middle-income countries can be leveraged for broader surveillance and diagnostic purposes. 8. Diversification in diagnostics manufacturing can make Global South countries less reliant on charity and enhance self-sufficiency. 9. Better tracking of diagnostics uptake and real-time sharing of data during Covid-19 can be extended to other conditions. 10. While diagnostics are now seen as a key component of effective pandemic preparedness, this can be expanded to ensure diagnostics are key for also universal health coverage.

Around the world, conversations over family dinner tables in the last few years included debates on different types of tests and test performance. Billions of people have undergone Covid testing. This unprecedented level of public engagement and intimate connection with testing could be used to demand better access to diagnostics for other conditions and increase use of testing to manage health conditions.

During the pandemic, political leaders were briefed daily on Covid-19 testing rates. This stands in contrast with other diseases where testing rates are reported months to years later, if at all. While there are other challenges for political attention in the current geopolitical and economic climate, the continuing focus on surveillance systems and pandemic preparedness in the political agenda creates an opportunity to ensure that diagnostics are seen as a global good by policy makers, and a key component of all country plans for university health coverage (UHC).^4^

With Covid-19 being ‘the first pandemic of the digital age’, there has been near universal deployment of digital tools to support all aspects of the response. Millions of people have used digital tools to get testing appointments as well as test results. While parts of this deployment have been haphazard, it is clear that digital integration with diagnostic tools will now be the standard, giving individuals more power over their own health decisions and strengthening data driven decision making for front-line health managers. LMICs must invest resources to overcome the digital divide, so the real potential of digital tools can be realized.^5^

Self-testing and home-based testing became normalized during the pandemic. Tests were administered at home, in schools and workplaces, giving people more control over their own health. This familiarity with self-testing, coupled with technology advances and clearer regulatory pathways, could be a game-changer for other diseases. This is especially true when stigma hinders access such as sexually transmitted infections, HIV or tuberculosis. Self-testing for airborne respiratory infections (e.g. Covid-19, flu, RSV) may also enable smarter decisions on self-isolation.

Many countries have seen a massive increase in the number of laboratories capable of undertaking molecular testing driven by the needs of Covid-19. This investment is a valuable platform that can be redeployed to help address other diseases, since molecular testing platforms are multi-disease. We need to take advantage of this ‘sunk cost’ during the emergency phase of the pandemic to drive better access to testing and to build from for more effective diagnostic and surveillance systems.

The pandemic has driven a rapid leap forward in the pipeline for miniaturized and simplified molecular platforms that can be decentralized and used in primary health clinics or even homes. The pipeline is now full of promising technologies, with FIND tracking more than 165 promising technologies. These devices could play a vital joint role in both improving clinical care and building effective surveillance systems through their data sharing capabilities. The fact that Asia is now a hotbed of diagnostic innovations^6^ bodes well for access for LMICs.

Before the pandemic, next generation sequencing (NGS) was seen as too complex for low-resource settings. However, the need to track SARS-CoV2 variants has pushed most countries, including countries in Africa, to invest in NGS.^7^ This high-tech tool is increasingly playing a central role in regular surveillance and we expect an acceleration in the timeline for its use in clinical care for tricky diseases such as drug resistant infections.

Poor access to products in Global South countries, and the breakdown in the convoluted and fragile diagnostics supply chain early in the pandemic exposed how concentrated manufacturing was in just a few countries. Supporting manufacturing of health technologies such as vaccines, medicines and diagnostics in the Global South has reached up the political agenda like never before. Diversification in diagnostics manufacturing is critical to make Global South countries less reliant on external support.

In fact, for its G-20 Presidency this year, India has identified access to safe, effective, quality, and affordable vaccines, therapeutics and diagnostics as key priority, with a huge emphasis on manufacturing in LMICs.^8^ Connecting this local or regional manufacturing and self-determination agenda with an access agenda as a common interest across ministries of trade & industry and ministries of health will be critical to realize the potential of this political agenda in driving wider access to diagnostic testing in LMICs.

Diagnostics stakeholders including industry, countries, procurers, and implementers came together like never before during the pandemic to share data, track development pipelines, support equitable allocation, track uptake and testing rates in near real time.^2^ This supported better coordination and targeting of limited resources. This end-to-end partnership to support innovation scale-up could be invaluable for our response in other key priorities in global health such as addressing testing gaps for diseases like HIV, TB, malaria or building surveillance systems.

Lastly, the pandemic exposed to policy makers how little they previously knew about their laboratory infrastructure, leaving many to urgently map and retool laboratory networks to respond to early waves of the pandemic. Consequently, diagnostics is now seen as a core component in strengthening future pandemic preparedness. While this is much-needed progress, it is critical to ensure that diagnostics are not just used during outbreaks. Any pandemic-related diagnostic investments must translate into better access to *all* essential diagnostics, since testing is critical to achieve the goal of UHC.^4^^,^^9^ UHC, in turn, is essential for pandemic preparedness and response. In this context, we welcome the upcoming resolution at the World Health Assembly, urging member states to strengthen diagnostics capacity.^10^

## Contributors

Both authors contributed equally to drafting and revising the Comment.

## Declaration of interests

The authors have no financial or industry-related conflicts. EH is an employee of FIND, a non-profit, global alliance for diagnostics. MP is an advisor to FIND & the Bill & Melinda Gates Foundation.
